# Phenotypic and Molecular Characterization of Plasmid Mediated AmpC **β**-Lactamases among *Escherichia coli*, *Klebsiella* spp., and *Proteus mirabilis* Isolated from Urinary Tract Infections in Egyptian Hospitals

**DOI:** 10.1155/2014/171548

**Published:** 2014-06-09

**Authors:** Mai M. Helmy, Reham Wasfi

**Affiliations:** ^1^Department of Microbiology & Immunology, Faculty of Medicine, Zagazig University, Zagazig, Sharqia,, Egypt; ^2^Department of Microbiology and Immunology, Faculty of Pharmacy, October University for Modern Sciences and Arts (MSA), 26 July Road Intersection with El Wahat Road, 6th of October, Giza, Egypt

## Abstract

The incidence of resistance by Enterobacteriaceae to **β**-lactam/**β**-lactamase inhibitors combination is increasing in Egypt. Three phenotypic techniques, comprising AmpC disk diffusion and inhibition dependent methods using phenylboronic acid (PBA) and cloxacillin, were compared to PCR based method for detection of plasmid mediated AmpC **β**-lactamase in common urinary tract isolates. A total of 143 isolates, including *E. coli*, *Klebsiella pneumonia*, and *Proteus mirabilis*, were collected from urinary tract infections cases in Egyptian hospitals. Plasmid encoded AmpC genes were detected by PCR in 88.46% of cefoxitin resistant isolates. The most prevalent AmpC gene family was CIT including CMY-2, CMY-4, and two CMY-2 variants. The second prevalent gene was DHA-1 which was detected in *E. coli* and *Klebsiella pneumonia*. The genes EBC, FOX, and MOX were also detected but in small percentage. Some isolates were identified as having more than one *pAmpC* gene. The overall sensitivity and specificity of phenotypic tests for detection of AmpC **β**-lactamase showed that AmpC disk diffusion and inhibition dependent method by cloxacillin were the most sensitive and the most specific disk tests. PCR remains the gold standard for detection of AmpC **β**-lactamases. This study represents the first report of CMY-2 variants of CMY-42 and CMY-102 **β**-lactamase-producing *E. coli*, *Klebsiella pneumonia*, and *Proteus mirabilis* isolates in Egypt.

## 1. Introduction

AmpC *β*-lactamases have gained importance for over than forty years, since their discovery, as one of the enzymes responsible for antimicrobial resistance in Gram-negative bacilli. AmpC *β*-lactamases are either plasmid or chromosomal mediated.* Citrobacter freundii*,* Enterobacter cloacae*,* Morganella morganii*,* Hafnia alvei*, and* Serratia marcescens* are organisms having chromosomally mediated AmpC *β*-lactamases. In late 1980s plasmid-borne AmpC cephalosporinases were detected that appear to be genetic descendants of the chromosomally encoded AmpC enzymes [1]. The presence of such genes on plasmids facilitated their spread between the family of Enterobacteriaceae as* Klebsiella* spp.,* Escherichia coli*,* Proteus mirabilis,* and* Salmonella* spp. [2]. The increased presence of plasmid mediated AmpC (*pAmpC*) *β*-lactamases worldwide is becoming of great concern [2]. AmpC *β*-lactamases are characterized by their ability to inactivate cephamycins in addition to other extended-spectrum cephalosporins and being resistant to clavulanic acid [3]. Infections caused by AmpC-positive bacteria are of particular clinical and epidemiological importance and cause higher patient morbidity and mortality [4, 5].

In Gram-negative organisms, the detection of AmpC-mediated resistance is problematic as phenotypic techniques may give misinterpreted results and, consequently, treatment failures [6]. Moreover, there are no guidelines of Clinical and Laboratory Standards Institute (CLSI) for phenotypic techniques to investigate AmpC-producing organisms [7].

A number of detection methods for AmpC *β*-lactamases have been proposed. These screening tools include resistance to cephamycins and/or ceftazidime [8], retaining cefepime susceptibility [9], modified cefoxitin Hodge test [10] and Tris-EDTA disc test [11], inhibitor-based assays (e.g., using boronic acid compounds [12] or cloxacillin [13]), and rapid chromogenic assays [14]. However these methods are not suitable for routine clinical use in clinical microbiology laboratories and for detection of all AmpC *β*-lactamases [15].

Polymerase chain reaction (PCR) analysis can be used to detect the presence of* AmpC* genes. Pérez-Pérez and Hanson [16] have detected six groups of plasmid mediated AmpC using PCR. These detected groups are ACC, DHA, CMY, EBC, FOX, and MOX. These groups were suspected to originate from* Hafnia alvei*,* Morganella morganii*,* Citrobacter freundii, Enterobacter cloaca, *and* Aeromonas *spp. [17].

There is increasing need for simple methods to detect the resistance mediated by* pAmpC* *β*-lactamase. The aim of this study was to (i) evaluate efficacy of different phenotypic methods compared to PCR as a gold standard test for rapid and accurate detection (ii) and to characterize the most prevalent gene encoding* pAmpC* enzymes.

## 2. Materials and Methods

### 2.1. Collection of Isolates

A total of 143 Gram-negative clinical isolates were collected from hospitalized cases of urinary tract infections from three Egyptian hospitals (Ain Shams, 6th of October, and Dar El Fouad hospitals). Collection was in the period from September 2011 to October 2012.

Isolates identification was based upon colonial characteristics and conventional biochemical tests [18]. The present study was approved by the Research Ethics Committee of the University and written consent was also taken from the patients.

The isolates were selected according to the following inclusion criteria: (i) species that are known to lack chromosomal AmpC (*Klebsiella* spp. and* P. mirabilis*) or are known to minimally express a chromosomal AmpC enzyme (*E. coli*) and (ii) isolates showing decreased susceptibility to cephamycins indicated by cefoxitin (FOX) intermediate or resistance phenotype. Isolates were screened by disk diffusion method according to the criteria published by the CLSI [19]. All antibiotic discs used were from Oxoid (Cambridge, UK).

### 2.2. Phenotypic Tests for Detection of ESBL

The phenotypic confirmation double disc synergy test (DDST) according to Dalela et al. [20] was used for detection of ESBL in the studied isolates.

### 2.3. Phenotypic Tests for Detection of AmpC

#### 2.3.1. Screening for Cefoxitin Resistant Isolates

Screening was done by the disc diffusion method of Lorian [21]. Isolates with clear zones, surrounding cefoxitin disc (30 *μ*g), of less than 18 mm were considered AmpC positive [22].

#### 2.3.2. Confirmatory Tests for AmpC *β*-Lactamase

*(a) AmpC Disc Test*. The test was done according to the method of Black et al. [11] using Cefoxitin-sensitive* E. coli *ATCC 25922 (supplied by Dar El-Fouad Hospital, 6th of October, Egypt).

*(b) Inhibitor-Based Methods for Detection of AmpC *β*-Lactamases*. Inhibitors include cloxacillin and phenylboronic acid and tests were done according to Thean et al. [12] and Manchanda and Singh [23].

### 2.4. Molecular Detection of* pAmpC *Genes

#### 2.4.1. Preparation of Template DNA

Plasmid DNA extraction and purification were done using the GeneJET Plasmid Miniprep Kit (Thermo scientific, Surrey, UK).

#### 2.4.2. PCR Protocol

The detection of the six different families of plasmid mediated AmpC *β*-lactamases including ACC, CIT, DHA, EBC, FOX, and MOX was done using the primers designed by Pérez-Pérez and Hanson [16]. All primers were synthesized and supplied by Fermentas (Carlsbad, Canada).

Two annealing temperatures were used including 54°C for amplification of genes belonging to the FOX and MOX families and 64°C for the other four genes families.

#### 2.4.3. Sequence Analysis of PCR Amplicon

Gene sequencing was done via Macrogen (Seoul, South Korea). The PCR products were sequenced using the Applied Biosystems Automated 3730XL DNA sequencer (Applied Biosystems, Foster City, CA, USA) and primers used in gene amplification. The sequences were edited in BioEdit Sequence Alignment Editor (http://www.mbio.ncsu.edu/BioEdit/page2.html) from Citeline (internet research software) according to the quality of the curves in the sequencing diagram. Sequence alignments and analyses were performed online using the BLAST program (http://www.ncbi.nlm.nih.gov/).

### 2.5. Data Analysis

The performances of various phenotypic test methods were evaluated by comparing their results to those of PCR method. Sensitivity and specificity for used phenotypic tests were calculated according to Lee et al. [24].

## 3. Results

Among the 143 isolates collected the isolates identification was* Escherichia coli* (102),* Klebsiella pneumoniae* (30),* Klebsiella oxytoca* (5),* Proteus mirabilis* (4), and* Proteus vulgaris* (2).

Twenty-six (18.2%) of 143 isolates were cefoxitin resistant. Of these isolates, 21* E. coli*, 3* K. pneumoniae*, one for* K. oxytoca,* and one* P. mirabilis* were thus considered as putative AmpC producers.

Among 21 cefoxitin resistant* E. coli* isolates, AmpC phenotype was confirmed in these isolates by AmpC disc, inhibitor based methods by cloxacillin, and phenylboronic acid testing, in 76.9% (*n* = 16), 76.9% (*n* = 16), and 66.6% (*n* = 14), respectively (Table 1). On the other hand, one isolate (1/3) of* Klebsiella pneumoniae* was confirmed by the three phenotypic methods as AmpC producers, while the* Klebsiella oxytoca* isolate was confirmed as AmpC producers only by the inhibitor based method using phenylboronic acid (Table 2). Moreover* Proteus mirabilis* isolate was confirmed phenotypically by the AmpC disc and the cloxacillin inhibition methods only (Table 2).

Among the 26 cefoxitin resistant isolates, plasmid encoded AmpC genes were detected by PCR in 23 (88.46%) isolates, which included* E. coli* (*n* = 19),* K. pneumoniae* (*n* = 2),* K. oxytoca* (*n* = 1), and* Proteus mirabilis* (*n* = 1).

**Figure 1:**
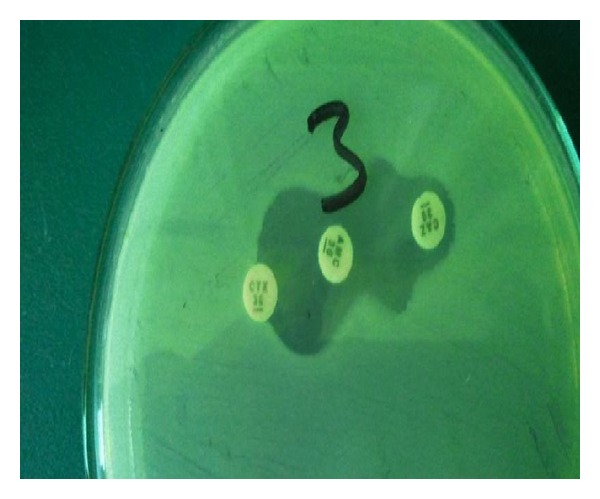
Double disc synergy test (DDST) for detection of ESBL production showing increase in zone of inhibition around ceftazidime (CAZ) and cefotaxime (CTX) towards augmentin disc (AGE).

The percentage of isolates showing positive ESBL by phenotypic methods was 80.7% (Figure 1). Two* E. coli* among these ESBL producing isolates did not have detectable p*AmpC* genes by all used methods except PBA.

**Figure 2:**
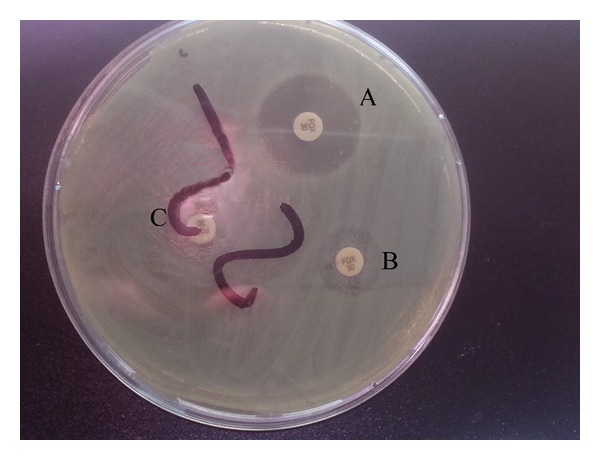
Inhibitor based method for detection of AmpC *β*-lactamase in isolate E2 with increase in zone of inhibition surrounding discs A: cloxacillin with cefoxitin, B: phenyl boronic acid with cefoxitin, and C: Cefoxitin.

**Figure 3:**
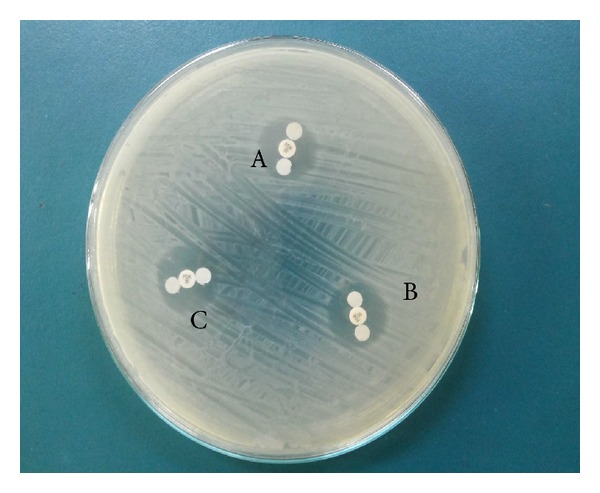
AmpC disc test. A: presence of blunting towards cefoxitin disc indicates positive test, B and C: absence of blunting indicates negative test.

The overall sensitivity and specificity of phenotypic tests for detection of AmpC *β*-lactamase showed that cloxacillin (Figure 2) and AmpC (Figure 3) were the most sensitive and the most specific disc tests (78.3% both, 100% both, resp.) followed by boronic acid disc test (65.2% and 73.9%, resp.). On comparing the inhibitory effect of cloxacillin and boronic acid on AmpC enzymes effects, it was noted that cloxacillin was a better inhibitor particularly among AmpC-positive* E. coli* and* P. mirabilis isolates* (Table 3) (Figure 2).

**Figure 4:**
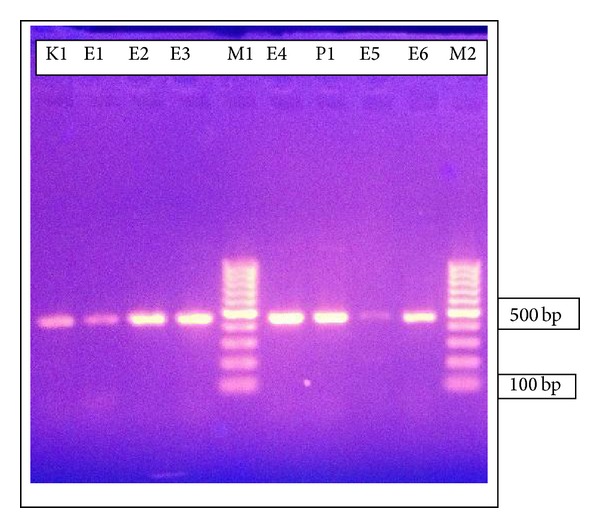
Agarose gel electrophoresis of AmpC *β*-lactamase genes amplified from eight different isolates. Lanes M1 and M2 are 100 bp DNA marker. Lanes from 1 to 4 and from 6 to 9 are the amplified product of CIT gene family (500 bp). The isolate K1 is* Klebsiella pneumoniae*, E1-6 are* E. coli* isolates, and P1 is* Proteus mirabilis* isolate. The size of the marker in base pairs is shown on the right.

The most prevalent p*AmpC* gene was that belonging to family CMY which was detected in 86.9% (20/23) isolates (Figure 4).

Eight isolates were found to carry more than one* AmpC* gene as shown in Tables 2 and 3.

Gene belonging to CMY family was the only one found in* Proteus mirabilis* (Table 3). No genes belonging to the ACC family were detected in all isolates.

*AmpC *nucleotide sequences retrieved from the 23 AmpC-positive isolates were analyzed to determine their relationship to other* pAmpC* genes available in GenBank database using the BLAST nucleotide algorithm (http://www.ncbi.nlm.nih.gov/).

Sequence analyses of PCR products from amplification of plasmid* AmpC* genes showed that the CMY genes from five* E. coli* isolates and* Klebsiella oxytoca* isolate were homologues to CMY-2 gene. CMY genes detected in eight* E. coli*, two* Klebsiella* spp.,* and Proteus mirabilis *isolates were 99%similar to CMY-42 gene (GenBank accession number HM146927.1) which is a CMY-2 variant. The CMY gene detected in the other three* E. coli* isolates showed 99% similarity to CMY-102 gene (GenBank accession number KF526115.1) which is another CMY-2 variant. One* E. coli* isolate was 99% homologues to CMY-4 (GenBank accession number GU056841.2). Two* E. coli* and one* Klebsiella pneumonia* isolates showed no AmpC gene belonging to the six known families.

Sequence analysis for PCR product showed that the genes of DHA family have 99% homology to DHA-1 gene (GenBank accession number HQ188691.1).

## 4. Discussion

High AmpC production level results in high clinical treatment failures with broad-spectrum cephalosporins [9]. The exact prevalence of AmpC *β*-lactamases is unknown and this may be due to the absence of simple and reliable detection methods in clinical laboratories.

The present study demonstrated that from the 26 cefoxitin resistant isolates about 23 were found to possess plasmid AmpC *β*-lactamase gene by PCR. This agreed with Fam et al. [25] and Yilmaz et al. [26] who found that not all cefoxitin resistant isolates are AmpC *β*-lactamase producers. This can be explained by the following. First, cefoxitin resistance is not only due to AmpC *β*-lactamase production, but also could be due to some other enzymatic mechanism as extended spectrum beta lactamases (ESBLs) and metallo beta lactamase (MBL) or nonenzymatic mechanism like porin channel mutation [27]. Second, cefoxitin resistant phenotype in* E. coli* can result from overexpression of the chromosomal* AmpC* gene due to mutations in the promoter and/or attenuator regions [28]. Third, cefoxitin has been demonstrated as a substrate to active efflux pump in clinical isolates [29].

Results of the present study showed that the prevalence of AmpC genes in collected isolates was 16.8%. The result was equivocal with studies carried by El-Hefnawy [30] and Fam et al. [25] from Egypt where AmpC prevalence was 34% and 28.3% respectively. The high prevalence of AmpC producers could be explained; as specimens were collected from inpatients and from patients admitted to ICU, it could be expected that they have been exposed to previous cephalosporin therapy whether empirically or according to the hospital antibiotic policy or due to unjustified courses as reported in a previous Egyptian study by El-Kholy et al. [31]. Knowing that selective pressure is produced by the extensive use of oxyimino-cephalosporins is among the driving forces of increasing the prevalence of AmpC-production [32].

False positive results encountered with phenotypic tests may be explained by the following. First is the possibility of presence of more AmpC *β*-lactamase genes that continue to expand beyond those contained in the six families genes covered by PCR [33]. Second, phenotypic tests are not able to differentiate between positive results due to upregulated chromosomally mediated AmpC *β*-lactamases and those due to genes that are carried on plasmids [12]. On the other hand, false-negative results may be explained by the fact that the genes may be detected by PCR but are not effectively phenotypically expressed.

In the current study, APB, cloxacillin, and AmpC disc tests were evaluated for detection of AmpC enzymes versus PCR. The current study showed that cloxacillin was a better inhibitor of AmpC enzymes than boronic acid as it gave higher sensitivity and specificity (78.3% and 100%, resp.). Boronic acid showed high rate of false positive results and this can be explained as boronic acid not only inhibits AmpC enzyme but also inhibits* Klebsiella pneumoniae* carbapenemases (KPC) enzyme [34]. False-negative results of APB in* E. coli* and* Klebsiella pneumonia* isolates that showed ESBL activity agreed with Brenwald et al. [13] study which noted that the activity of ESBL masked the inhibitory effect of boronic acid utilized in their study. Coudron [35] suggested that swarming phenomena may interfere with ability of boronic test to detect* pAmpC* producing* P. mirabilis* as was noticed in our study.

AmpC disk test (Tris-EDTA test) gave the best result in this study (sensitivity 78.3% and specificity 100%). Thomson [34] explained that by the fact that Tris-EDTA improves the release of *β*-lactamases by permeabilizing Gram-negative cells and its inhibition of MBL carbapenemase activity improved the specificity of the test by preventing cefoxitin hydrolysis by this enzyme.

No ACC family genes were detected; this can be explained by the fact that the enzymes of this family appear to be inhibited by cefoxitin [36, 37] and in this study all isolates used were cefoxitin resistant. This agreed with Thean and his colleagues [12] who found that screening for cephamycin-resistance is a less sensitive tool for the ACC family of enzymes. Hosny and Kashif [40].

The geographical area, the species studied, and the period of study influence the prevalence and type of acquired* pAmpC*s detected [38, 39]. For this reason, it is difficult to compare the prevalence of acquired AmpCs between studies.

In the present study, amplification of DNA by PCR using different primers for* pAmpC* clusters revealed that CMY homologues was the most predominant gene (86.9%) followed by DHA (21.7%), FOX (17.3%), EBC (13%), and MOX (13%). These results were in agreement with two studies carried in Cairo, Egypt, by Fam et al. [17] and Hosny and Kashif [40] in which CMY was detected in 76.5% and 60% while DHA-1 in the 23.5% and 40%, respectively. On the other hand, in studies carried by Barwa et al. [41] and Wassef et al. [42], the FOX family showed the highest prevalence rate.

It was noticed in this study that some of the* E. coli* and* Klebsiella pneumoniae isolates* harbored more than one AmpC gene family and that was reported in several studies [43, 44].

The amplified portion of CMY-genes detected in this study includes homologues of CMY-2, CMY-4, and two CMY -2 variants (CMY-42 and CMY-102) with percentage of similarity 99%. DNA sequence analysis showed that CMY-2 gene and its alleles were found in 15/19 of* pAmpC*-positive* E. coli* isolates. DHA-1 was detected in two isolates of* E. coli*. These findings were consistent with previous studies confirming* pAmpC* *β*- lactamases of the CMY-2 type in* E. coli* as a major factor contributing to AmpC resistance phenotype [28, 41, 45]. The* bla*CMY-2 gene is the most prevalent and has been reported worldwide. Interestingly,* bla*CMY-2 is found in many different plasmid backgrounds, suggesting that it can be mobilized as a part of a smaller transferable fragment [46].

The widespread distribution of plasmid CMY-2 among* Enterobacteriaceae* could be attributed to specific transposon-like element IS*Ecp1* [47, 48]. The IS*Ecp1* has been presumed to be involved in the mobilization of* bla*CMY-2 from the* Citrobacter freundii* chromosome [48].

In Egypt, the presence of CMY-4 gene cluster was previously reported in a study carried by Fam et al. [17] in* Klebsiella* spp. isolate but in our study it was detected in a single* E. coli* isolate. CMY-4 was first identified in a* P. mirabilis* isolate from Tunisian patient and is also believed to originate from the chromosome of* C. freundii* [49].

Overexpression of AmpC yields clinical resistance to virtually all *β*-lactam agents, with the possible exception of imipenem and meropenem, and so remains the best treatment option in treating serious infections caused by* pAmpC*-producing isolates even in case of coproduction of ESBL enzymes [19]. Detecting* pAmpC* isolates may be clinically important not only because of their broader cephalosporin resistance but also because carbapenem resistance can arise in such strains by further mutations, resulting in reduced porin expression [50, 51].

DHA-1 is a plasmid mediated AmpC *β*-lactamase that originated from the chromosomal* AmpC* gene of* M. morganii* [52]. It has been shown in study carried by Pai et al. [53] and Moland et al. [54] that mortality of patients infected with organisms that produce DHA-1 was higher than that of patients infected with organisms that produce CMY-1 and that raises concern for the spread of this inducible plasmid mediated AmpC *β*-lactamase.

To our knowledge, this is the first report from Egypt recording homologues to CMY-4 in* E. coli* isolate; also it is the first recording homologues for the CMY-2 variants (CMY-42 and CMY-102) in Enterobacteriaceae isolates.

## 5. Conclusion

PCR is the gold standard method for detection of AmpC *β*-lactamase. Isolates of* E. coli, K. pneumoniae,* and* Proteus mirabilis* showed the occurrence of plasmid mediated AmpC *β*-lactamase which is alarming. The dissemination of these plasmid mediated resistance genes within the hospital or between the different regions of the country by conjugation may become an important public health issue. The most prevalent AmpC gene belongs to CMY-2 and CMY-2 variants followed by DHA-1. Hence, identification of types of AmpC may help the physician to prescribe the most appropriate antibiotic, thus decreasing the selective pressure, which generates antibiotic resistance.

## Figures and Tables

**Table 1:** Comparison of phenotypic and genotypic methods for detection of *pAmpCβ*-lactamase in cefoxitin resistant *E. coli* isolates.

		Phenotypic test			
		ESBL	AmpC disc test	Inhibitor-based methods using	
*AmpC *gene family	Number of isolates			Cloxacillin	Phenylboronic acid
MOX	2	+ve	+ve	+ve	−ve
FOX	1	−ve	−ve	−ve	−ve
CIT	1	−ve	+ve	+ve	+ve
CIT	7	+ve	+ve	+ve	+ve
CIT	2	+ve	+ve	+ve	−ve
CIT-DHA	2	+ve	+ve	+ve	+ve
CIT-DHA	1	−ve	+ve	+ve	+ve
CIT-FOX	1	+ve	+ve	+ve	+ve
FOX-MOX	1	+ve	+ve	+ve	−ve
—	2	+ve	−ve	−ve	+ve
CIT-EBC	1	+ve	+ve	+ve	−ve

**Table 2:** Comparison of phenotypic and genotypic methods for detection of plasmid mediated AmpC *β*-lactamase in cefoxitin resistant *Klebsiella* spp. and *Proteus mirabilis *isolate.

Microorganisms	*AmpC* gene family	Number of isolates	Phenotypic tests			
			ESBL	*bla* AmpC		
				AmpC disc test	Inhibitor-based methods	
					Cloxacillin	Phenylboronic acid
*Klebsiella pneumoniae *	CIT	1	+ve	−ve	−ve	+ve
*Klebsiella pneumoniae *	CIT-EBC-MOX	1	+ve	−ve	−ve	−ve
*Klebsiella oxytoca *	CIT-DHA-FOX	1	+ve	−ve	−ve	+ve
*Klebsiella pneumoniae *	—	1	−ve	+ve	+ve	−ve
*Proteus mirabilis *	CIT	1	−ve	+ve	+ve	−ve

**Table 3:** Comparison of sensitivities, specificities, and predictive values of phenotypic tests for detection of AmpC-positive isolates.

	Cloxacillin test	AmpC disc test	Phenylboronic acid
Sensitivity	78.3%	78.3%	65.2%
Specificity	100%	100%	73.9%
PPV*	100%	100%	94.4%
NPV^†^	37.5	37.5	20%

Notes:*indicates positive predictive value.

^†^indicates negative predictive value.
